# Swimming turns reduce energy demands of the aerobic performance in front crawl

**DOI:** 10.1007/s00421-025-06084-7

**Published:** 2025-12-06

**Authors:** Pietro Bosetto, Vittorio Coloretti, Myriam Lubrano, Marco Bonifazi, Paola Zamparo, Silvia Fantozzi, Matteo Cortesi

**Affiliations:** 1 Department for Life Quality Studies, University of Bologna, Bologna, Italy; 2 Department of Electrical, Electronic, and Information Engineering “Guglielmo Marconi”, University of Bologna, Bologna, Italy; 3 Interdepartmental Center for Industrial Research on Health Sciences and Technologies, University of Bologna, Bologna, Italy; 4 Department of Medical Biotechnologies, University of Siena, Siena, Italy; 5 Department of Neuroscience, Biomedicine and Movement Sciences, University of Verona, Verona, Italy

**Keywords:** Energy cost, Pool length, Short-course swimming, Long-course swimming, Oxygen uptake

## Abstract

**Purpose:**

Performances in short-course (SC, 25 m) are typically faster than in long-course (LC, 50 m), largely due to the greater number of turns, but the specific energetic contribution of turns has not yet been quantified. This study tested the hypothesis that turns reduce the overall energy cost (C) in swimming, providing an energetic advantage in SC over LC.

**Methods:**

Eleven male swimmers completed two randomized sessions in SC and LC pools, each consisting of five 400-m front crawl trials at submaximal intensity (70–86% of race velocity) paced by an underwater light system. Turn and clean swimming velocities were standardized between conditions to isolate the effect of turn number. Oxygen uptake, blood lactate, heart rate, perceived exertion, and stroke frequency were assessed, and metabolic power, total energy expenditure (E_tot_), and C (E_tot_/distance) were calculated.

**Results:**

When analyzed at equivalent intensity (e.g. in trials corresponding to the same % of race velocity) mean velocity was higher in SC than LC across all intensities (+ 0.07 ± 0.003 m·s⁻¹, + 5.2%) while kinematic, physiological, and energetic parameters showed no significant differences (*p* > 0.05). When analyzed at paired (absolute) speeds, C values were about 4% higher il LC than in SC, indicating that swimming in short course is more economical, as hypothesized.

**Conclusions:**

Turns reduce the overall energy cost of 400-m front crawl performance enabling swimmers to sustain higher mean velocities in SC. This highlights the importance of considering pool length when evaluating performance and prescribing training intensities.

## Introduction

The mean velocity of a swimmer (v_mean_) is determined by the ratio between metabolic power (Ė) and energy cost (C): v_mean_ = Ė/C where C is the metabolic energy required to cover a unit distance (or the metabolic power needed to sustain a given velocity) (di Prampero et al. 1986; Zamparo et al. 2020). Hence, for a given metabolic demand (Ė) a faster swimming velocity can be achieved by reducing C.

A substantial proportion of swim training is performed at submaximal intensities, with workloads below the lactate threshold representing up to 90% of total training volume (Hellard et al. 2019). In this domain, Ė is sustained by aerobic energy sources (Ė = V̇O₂), (di Prampero et al. 1986; Zamparo et al. 2020). As race duration increases, Ė corresponds to the fraction of maximal oxygen uptake that can be sustained over time (Brueckner et al. 1991; Jones et al. 2021).

For a given race distance performed at submaximal intensity, v_mean_ differs between short-course (25 m; SC) and long-course (50 m; LC) competitions because v_mean_ depends on starting velocity (v_start_), clean swimming velocity (v_clean_), and turning velocity (v_turn_), and thus, also by the number of turns (Gonjo and Olstad 2021; Born et al. 2021). The turn segment contributes to a reduction in total race time (t_tot_) due to the increased velocity generated by the wall push-off (Polach et al. 2021). As a result, SC performances are typically ~ 2% faster than LC ones (Wolfrum et al. 2013).

With increasing race distance, the relative time contribution of turns to v_mean_ increases and can exceed 30% of total swimming time (Polach et al. 2021), underscoring the need to quantify their energetic impact. Despite their relevance, the specific effect of turns on swimming energy demands has been largely neglected, also because measuring oxygen uptake (V̇O₂) in the aquatic environment poses technical challenges (e.g. Zamparo et al. 2020). Indeed, most studies assess aerobic energy expenditure (E_ae__r_) under open-turn conditions (without the underwater phase and the push off from the walls) because swimmers must be connected to a metabolimeter via a snorkel (Monteiro et al. 2020). Clearly, these protocols deviate substantially from race and training conditions (Sousa et al. 2014). To overcome this constraint, the back-extrapolation approach can be utilized: it estimates V̇O₂ from end-exercise measurements (e.g. Zamparo et al. 2020). However, when using back-extrapolation, caution is needed due to the rapid post-exercise decline of V̇O₂, which may limit its accuracy (Sousa et al. 2014).

To date, the only SC–LC comparison of v_mean_ and physiological variables is by Keskinen et al. (2007), who asked trained swimmers to perform two incremental 5 × 200 m front-crawl trials at the same percentage of velocity. The authors concluded that LC swimming was more physiologically demanding, as evidenced by higher La^−^ concentrations, increased HR at submaximal intensities, and lower v_mean_ compared to SC. However, their protocol did not isolate the specific contributions of turns to overall performance, limiting inference about the energetic role of turns. Accordingly, the present study aimed to quantify the energetic effect of turns on front crawl performance under submaximal conditions. By prescribing identical clean-swimming and turn velocities across SC and LC, the number of turns was isolated as the only differentiating factor. We hypothesized that, at equivalent physiological loads, SC would allow higher v_mean_ than LC due to a lower overall energy cost (C).

## Materials and methods

### Participants

Eleven males participated in the study. All participants were regional or national level competitive swimmers. Anthropometric, training characteristics and best performance time in the 400 m front crawl, expressed in seconds (s) and World Aquatics (WA) points are summarized in Table 1. All participants were informed about the methods and aims of the study. Participation was voluntary, and informed written consent was obtained from participants. The study was conducted in adherence to the Declaration of Helsinki and the procedure was approved by the local Ethics Committee of the University of Bologna (Approval code: 0312138, 10 October 2024).

**Table 1:** Anthropometric, training and performance characteristics of participants

Participants	Sex	Age (years)	Body Mass (kg)	Stature (cm)	Training/week	LC 400 m best time (s)	WA Points
1	M	32	82	193	8	239	781
2	M	18	88	190	6	263	586
3	M	28	75	184	8	243	743
4	M	23	71	177	6	255	643
5	M	26	80	178	8	260	607
6	M	23	73	180	8	255	643
7	M	23	79	182	8	268	554
8	M	22	73	189	9	260	607
9	M	25	77	182	5	263	586
10	M	26	78	180	6	249	666
11	M	20	71	176	6	271	536
Mean	/	24	77	183	7	257	632
SD	/	4	5	6	1	10	75

### Experimental design

This observational investigation employed a randomized, controlled-velocity protocol. Each swimmer completed two sessions, performed in random order, in two pool course configurations: short-course (25 m, SC) and long-course (50 m, LC). For each session, the swimmers performed five 400-m front crawl trials at an even pace within each trial, assisted by an in-lane light system, with the pace progressively increasing across trials; each trial was separated by 5 min of rest. The maximal velocity of the 400 m trial (v_100%mean_) was anchored to 96% of the swimmer’s long-course 400 m personal-best time (Table 1) as v_100%mean_ = 400 / (0.96 · best time), with this 4% adjustment reflecting the specific experimental training conditions (Houmard and Johns 1994). Five submaximal swim paces (v_70%mean_, v_74%mean_, v_78%mean_, v_82%mean_ and v_86%mean_) corresponding to 70, 74, 78, 82, and 86% of v_100%mean_ were repeated in SC and LC. These speeds were held constant in both SC and LC sessions, ensuring that the only experimental difference between conditions was the number of turns. Kinematic, energetic, and physiological variables were collected for SC-LC comparison. Testing was completed within a week (in SC and LC) and sessions were separated by ≥ 48 h. Swimmers avoided caffeine and strenuous exercise for 24 h before the tests and performed a 1000 m low-to-moderate warm-up before starting the experiments.

### Virtual pacing setup

To determine the controlled velocities for the underwater pacing system, each swimmer performed a 100 m trial at v_100%mean_ as part of the warm-up. During these warm-up trials the turn time (t_turn_) and the underwater distance covered during the turn (s_turn_) were measured using a calibrated sagittal-plane video camera (Panasonic, HC-V770, Kadoma; Japan). These parameters were used to calculate turn velocity as: v_turn_ = s_turn_ / t_turn_.

**Fig. 1:**
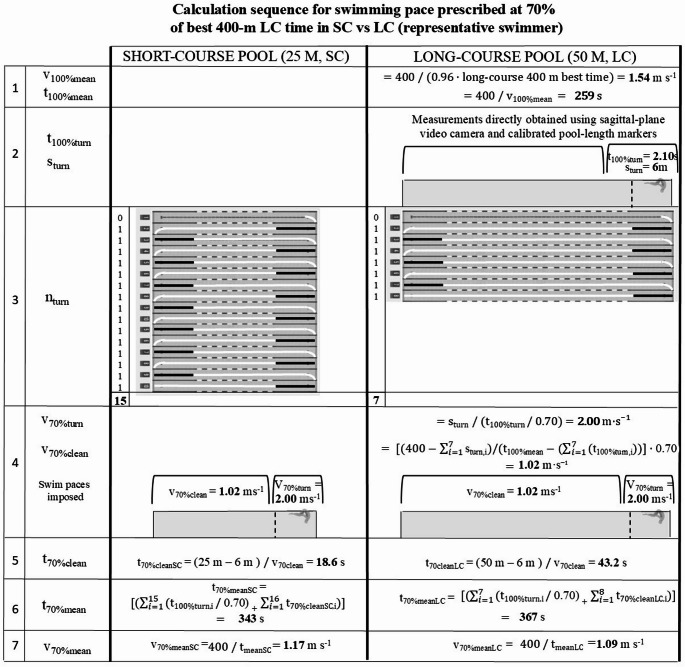
Example of the experimental protocol for Participant n.10 at a swim pace corresponding to 70% of his 96% LC best time, under controlled-velocity conditions in short-course (SC) and long-course (LC) pools. v_100mean_: mean swimming velocity at 96% of long-course 400 m best time; t_100%mean_: time of a single 400 m during v_100%mean_; t_100%turn_: time of a single turn during v_100%mean_; s_turn_: distance of a single turn; n_turn_: number of a turns; v_70%turn_: mean velocity of the turn during v_70%mean_; v_70%clean_: mean swimming velocity of a single 400 m excluding turn during v_70%mean_; t_70%clean_: time of a single 400 m excluding turns time during v_70%mean_; t_70%cleanSC_: time of a single 400 m in SC excluding turns time during v_70%mean_; t_70%cleanLC_: time of a single 400 m in LC excluding turns time during v_70%mean_; t_70%meanSC_: time of a single 400 m in SC during v_70%mean_; t_70%meanLC_: time of a single 400 m in LC during v_70%mean_; v_70%mean_: mean swimming velocity of a single 400 m during 70% of v_100%mean_; v_70%meanSC_: mean swimming velocity of a single 400 m in SC during 70% of v_100%mean_; v_70%meanLC_: mean swimming velocity of a single 400 m in LC during 70% of v_100%mean_

To calculate v_clean_ for the 400 m trials, total turn distance and total turn time for each trial were obtained by considering the number of turns (n_turn_); v_clean_ was then computed as: (400 - total turn distance) / (t_mean_ - total turn time). An example of this calculation sequence is reported in Fig. 1.

**Fig. 2:**
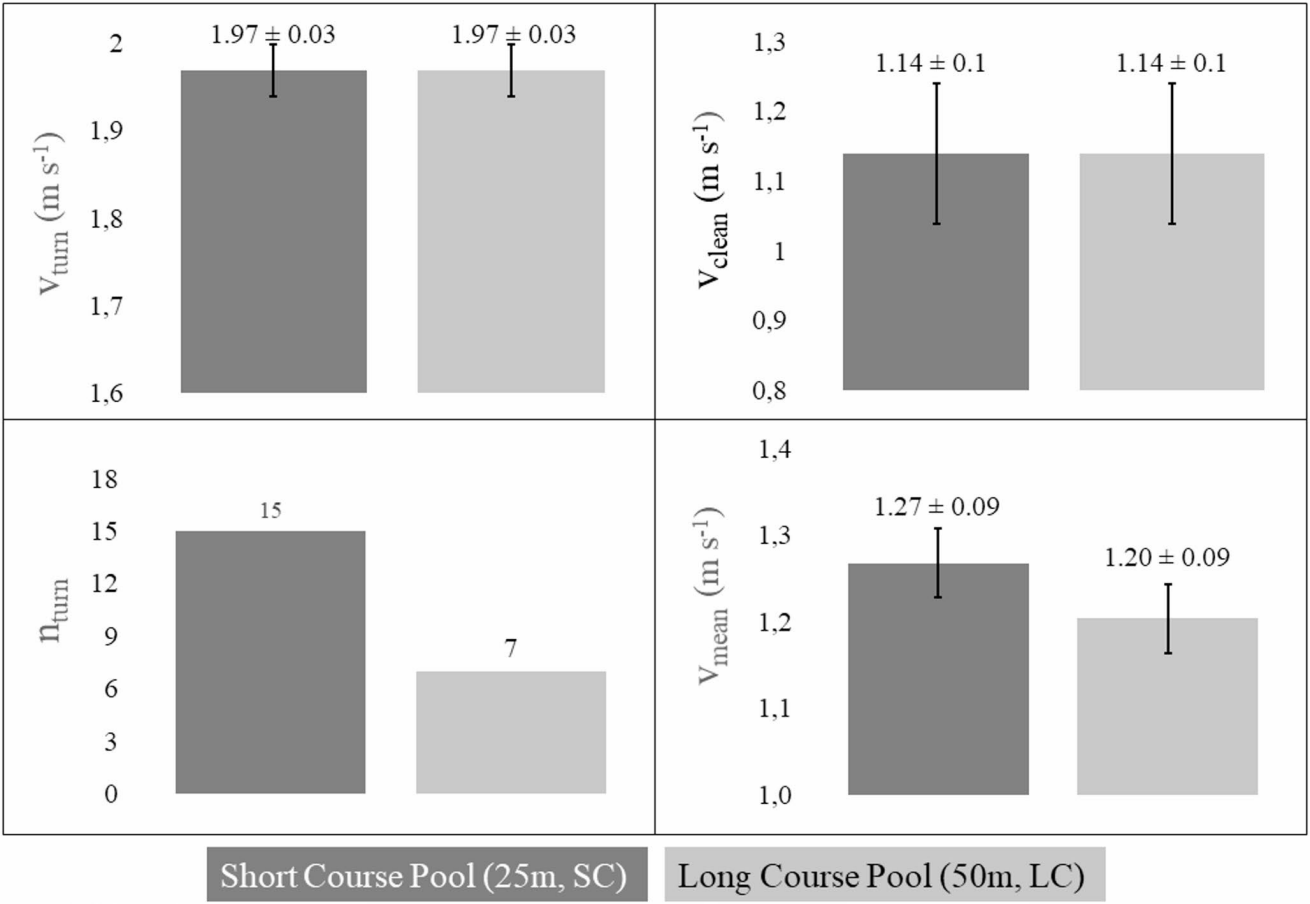
Comparison of mean ± SD variables between short-course (SC) and long-course (LC) during 5 × 400 m protocols at pre-set constant velocities determined by the in-lane light pacing system. v_turn_: mean turn velocity; v_clean_: mean swimming velocity excluding turn; n_turn_: number of turns; v_mean_: mean swimming velocity

An underwater LED pacing line strip (Indico Technologies, Turin, Italy), adjustable for 25–50 m pools was mounted on the pool floor and programmed to control v_turn_ and v_clean_ as pre-calculated for each swim pace. The v_turn_ was, on average, greater than v_clean_ by 0.83 ± 0.05 m·s⁻¹; consequently, mean velocity in short course (v_meanSC_) was higher than in long course (v_meanLC_) by 0.07 ± 0.003 m·s⁻¹, given to the greater number of turns (see Fig. 2).

### Kinematic, energetic, and physiological assessments

Stroke frequency (SF) was measured over five stroke cycles taken at mid-pool in both SC and LC conditions. Oxygen uptake (V̇O₂) was measured breath-by-breath using a portable metabolic system (K5, Cosmed, Rome, Italy) during the first 20–30 s of recovery at the end of each submaximal trial (for further details, see Chaverri et al. 2016; di Prampero et al. 1976, 1986; Montpetit et al. 1981; Rodríguez et al. 2000, 2015; Zamparo et al. 2000, 2005). Net oxygen uptake at steady state (V̇O₂_net_) was calculated as V̇O₂_net_ = V̇O₂ - V̇O₂_rest_ where V̇O₂_rest_ is the breath-by-breath resting value. Aerobic energy demand (E_aer_) for each trial was computed as E_aer_ = V̇O₂_net_ × t_%_ × 0.0209 assuming an energy equivalent of 20.9 kJ·L⁻¹O₂ (Capelli et al. 1999; Zamparo et al. 2000, 2020). Blood lactate concentration (La⁻) was measured via fingertip capillary sampling at 1, 3, 5, and 7 min after each trial to identify the peak value. Net lactate accumulation (La^−^ _net_) was calculated as the difference between post-exercise La⁻ and resting values (La⁻_rest_). Anaerobic lactic energy (E_anL_) was estimated as E_anL_ = βLa^−^ _net_ assuming an energy equivalent of lactate (β) of 2.7 ml O₂ kg⁻¹ mM⁻¹ and converted to kJ using an oxygen equivalent of 20.9 kJ·L⁻¹O₂ (di Prampero et al. 1981). Total energy expenditure (E_tot_) was then obtained as the sum of the aerobic and anaerobic contributions (E_tot_ = E_aer_ + E_anL_) (Zamparo et al. 2011, 2020). Finally, C was calculated as the ratio between E_tot_ and the total distance covered (400 m) (Zamparo et al. 2011, 2020). Heart rate (HR) was continuously monitored using an optical sensor (OH1+, Polar, Kempele, Finland) positioned at the temple under the swim cap, and averaged over the final minute of each trial. After each trial, the rating of perceived exertion (RPE) was recorded using the CR10 scale (Borg 1985).

### Statistical analysis

A statistical power analysis was performed to determine the required sample size using G*Power 3.1.9.7. By assuming an effect size of 0.40, an α error probability of 0.05, and a power (1–β error probability) of 0.80, the resulting total sample size required to achieve the desired statistical power was 10 participants. The normality of the data was tested using the Q-Q plot. A descriptive analysis including mean and standard deviation (SD) was carried out for all variables. The dependent variables (t_mean,_ v_mean,_ SF, V̇O₂_net,_ E_aer,_ E_anL,_ E_tot,_ C, RPE and La⁻) were compared using analyses of variance (ANOVAs) for repeated measures to investigate the effects of pool course (SC and LC) and swim intensity (70, 74, 78, 82 and 86% of v_100%mean_). In the case of a significant F ratio, a Bonferroni post hoc test was used to determine pairwise differences between conditions. For HR, the same comparison was conducted using a linear mixed model (LMM) to account for unpaired missing data. Differences in C at each 0.01 m·s⁻¹ increment of velocity (from 1.09 to 1.38 m·s⁻¹) between SC and LC were assessed based on the C vs. v relationship as determined in the two conditions. The significance level was set at *p* < 0.05. As reported by Ferguson (2009), the effect size (η²) was interpreted as: trivial if 0 < η² < 0.04, small if 0.04 ≤ η² ≤ 0.24, moderate if 0.25 ≤ η² < 0.64, and large if η² ≥ 0.64.

## Results

### Effect of pool course

When analyzed at equivalent intensity (e.g. in trials corresponding to the same % of race velocity), t_meanSC_ was significantly lower compared to t_meanLC_ across all tested conditions (*p* < 0.01, η² = 0.853), with a significant main effect of pool course (*p* < 0.01, η² = 0.140). Consequently, v_meanSC_ was significantly higher than v_meanLC_ at all intensities (*p* < 0.001, *η*^2^ = 0.856).

Stroke frequency (SF) did not differ between pool courses (*p* = 0.176, *η²* = 0.009). No significant differences in V̇O₂_net_ were observed between LC and SC (*p* = 0.120, *η²* = 0.046), and no interaction between pool course and intensity was found (*p* = 0.379, *η²* = 0.010). Aerobic (E_aer_, *p* = 0.493, *η²* = 0.014), anaerobic lactic (E_anL_, *p* = 0.343, *η²* = 0.008), and total energy contributions (E_tot_, *p* = 0.516, *η²* = 0.012), as well as the overall energy cost of swimming (C, *p* = 0.535, *η²* = 0.011), did not differ significantly between SC and LC. Blood lactate concentration (La⁻, *p* = 0.995, *η²* = 6.113 × 10⁻⁷) and rating of perceived exertion (RPE, *p* = 0.164, *η²* = 0.014) were unaffected by pool course. Heart rate (HR) showed a small but significant difference between SC and LC (*p* = 0.045). Mean and SD values of all analyzed variables (at equivalent intensities) are presented in Table 2.

**Table 2:** Mean values (± SD) of the kinematic, physiological, and energetic variables included in the analysis

	v70%		v74%		v78%		v82%		v86%	
	SC	LC	SC	LC	SC	LC	SC	LC	SC	LC
t_mean_ (s)	346 ± 11	368 ± 13***	330 ± 11	349 ± 12***	316 ± 10	333 ± 11***	303 ± 9	317 ± 11***	291 ± 9	304 ± 10***
t_mean_ (min)	5.77 ± 0.19	6.13 ± 0.21***	5.50 ± 0.18	5.82 ± 0.20***	5.26 ± 0.17	5.54 ± 0.19***	5.04 ± 0.16	5.29 ± 0.18***	4.84 ± 0.15	5.06 ± 0.18***
v_mean_ (s)	1.16 ± 0.04	1.09 ± 0.04***	1.21 ± 0.04	1.15 ± 0.04***	1.27 ± 0.04	1.20 ± 0.04***	1.32 ± 0.04	1.26 ± 0.04***	1.38 ± 0.04	1.32 ± 0.05***
SF (cyc min^− 1^)	23 ± 2	22 ± 1	24 ± 2	23 ± 2	25 ± 2	25 ± 2	27 ± 2	26 ± 2	28 ± 3	28 ± 2
V̇O₂_net_ (ml min^−1^)	2754 ± 527	2469 ± 713	3142 ± 530	2893 ± 787	3494 ± 645	2953 ± 763	3845 ± 747	3691 ± 762	4147 ± 409	3949 ± 826
V̇O₂ _net_ (ml kg^−1^ min^−1^)	36 ± 7	32 ± 9	41 ± 7	38 ± 10	47 ± 9	39 ± 10	51 ± 10	48 ± 10	55 ± 8	51 ± 11
E_aer_ (kJ)	331 ± 60	314 ± 84	361 ± 59	350 ± 88	383 ± 66	341 ± 85	405 ± 77	408 ± 84	419 ± 55	417 ± 83
E_anL_ (kJ)	4 ± 3	3 ± 4	2 ± 3	2 ± 2	3 ± 2	4 ± 3	6 ± 4	7 ± 3	12 ± 6	15 ± 6
E_tot_ (kJ)	335 ± 61	318 ± 86	363 ± 60	353 ± 88	386 ± 67	345 ± 86	410 ± 80	414 ± 85	431 ± 59	431 ± 85
Ė _tot_ (kJ s^− 1^)	0.97 ± 0.19	0.87 ± 0.25	1.10 ± 0.19	1.01 ± 0.28	1.23 ± 0.23	1.04 ± 0.24	1.36 ± 0.27	1.31 ± 0.27	1.49 ± 0.22	1.42 ± 0.30
La^−^ (mmol L^− 1^)	1.9 ± 0.9	1.7 ± 0.9	1.6 ± 0.8	1.5 ± 0.6	1.8 ± 0.6	1.9 ± 0.8	2.4 ± 0.8	2.5 ± 0.8	4.0 ± 1.2	4.3 ± 1.7
RPE (CR10)	1 ± 1	1 ± 0	2 ± 1	1 ± 1	3 ± 1	2 ± 1	3 ± 1	3 ± 1	5 ± 1	4 ± 2
HR (bpm min^− 1^)	129 ± 10	125 ± 10	137 ± 13	131 ± 12	148 ± 13	142 ± 11	158 ± 12	155 ± 11	169 ± 10	167 ± 10
C (kJ m^− 1^)	0.84 ± 0.15	0.79 ± 0.21	0.91 ± 0.15	0.88 ± 0.22	0.97 ± 0.17	0.86 ± 0.22	1.03 ± 0.20	1.04 ± 0.21	1.08 ± 0.15	1.08 ± 0.22

***Bonferroni Post Hoc Comparisons (intensity * course) at matched intensity; *p* < 0.001

### Effect of swim pace

The t_mean_ differed significantly across swimming intensities (*p* < 0.01, η² = 0.853), with corresponding differences in v_mean_ (*p* < 0.001, η² = 0.856) between SC and LC.

The SF increased significantly with swim pace (*p* < 0.01, η² = 0.806). V̇O₂_net_ increased significantly with pace (*p* < 0.01, η² = 0.596). E_aer_ (*p* < 0.01, η² = 0.362), E_anL_ (*p* < 0.01, η² = 0.700), E_tot_ (*p* < 0.01, η² = 0.400), and C (*p* < 0.01, η² = 0.403) all increased with swim pace. La⁻ increased significantly with swim pace (*p* < 0.001, η² = 0.650), as did RPE (*p* < 0.01, η² = 0.764) and HR (*p* < 0.01).

**Fig. 3:**
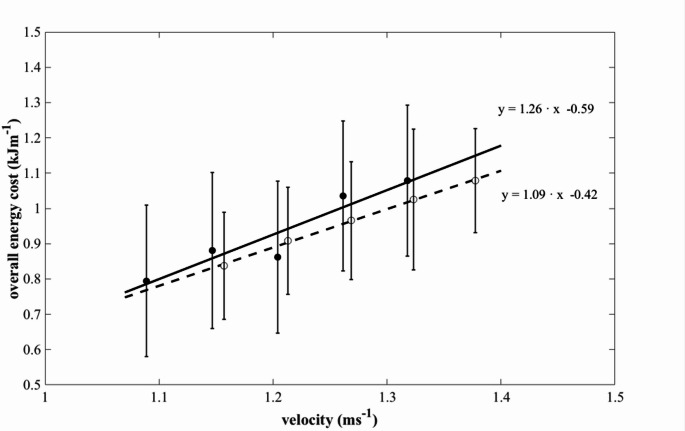
Overall Energy Cost (C) plotted for long-course (black symbols and continuous line) and short-course (white symbols and dotted line) across the range of pre-set swimming velocities used in this study. Data are means ± SD

In Fig. 3 the energy cost of swimming is reported as a function of the absolute (mean) swimming speed (data points are the average values, for all swimmers, at each exercise intensity and bars represent the standard deviation). This figure indicates that the increase in speed in SC is associated to a decrease in the energy cost of swimming, and hence that SC is more economical. Based on the linear equations relating C and v in the two conditions, the average difference in energy cost at paired speeds (at each 0.01 m·s⁻¹ increment of velocity, from 1.09 to 1.38 m·s⁻¹) can be calculated: it is comparable to the average difference in swimming speed (about 4%).

## Discussion

This study aimed to examine the influence of the turn segment on overall C of aerobic swimming performance and its implications. Our findings support the hypothesis that, for the same distance (400 m) covered at matched mean velocity, the increase in velocity in SC is associated to a reduction in the overall energy cost compared with LC, owing to the approximately twofold greater number of turns. Conversely, at equivalent exercise intensities (e.g. in trials corresponding to the same % of race velocity), v_mean_ is higher in SC than in LC conditions.

Turns represent a phase of reduced active swimming, and their relative contribution to overall performance is influenced by pool length. Specifically, the proportion of total time spent in turns is approximately twice as high in SC compared to LC competitions (Keskinen et al. 2007; Polach et al. 2021). As v_turn_ tipically exceeds v_clean_, overall performance is consistently faster in SC than in LC, a finding well established in the literature. Race analyses have shown SC times to be ~ 2.0 ± 0.6% faster than in LC (Born et al. 2021; Wolfrum et al. 2013), whereas in the present study the difference was greater (5.2%). The discrepancies between our findings and previous reports may be partly explained by the experimental constraint of performing turns in standardized conditions throughout the trial, a condition that does not fully replicate the ecological variability of competitive swimming. Indeed, during LC competitions, v_turn_ tends to remain stable throughout the race, whereas in SC it declines with increasing distance (Polach et al. 2021). Similarly, s_turn_ in SC progressively decreases, thereby reducing the propulsive advantage of the push-off (Keskinen et al. 2007; Polach et al. 2021). As a result, v_mean_ remains more stable in LC than in SC. Differences between published values and our results likely reflect that, in the present protocol, both v_clean_ and v_turn_ were experimentally determined (imposed).

In the present experimental protocol v_clean_ was imposed whereas stroke frequency was self-selected. Since swimming velocity is determined by the product of SF and stroke length, and swimmers predominantly regulate velocity through adjustments in SF (Takagi et al. 2023), this approach allowed participants to modulate their stroke mechanics while maintaining the target v_clean_. In our study, no difference in SF between pool courses was observed at a given effort. It therefore appears that, although SC and LC differed substantially in the number of turns and uninterrupted clean-swimming duration (≈ 19 vs. ≈ 43 s; Fig. 1), the average SF was not different between pool courses. These findings indicate that, when v_clean_ and v_turn_ are controlled, pool course per se (25–50 m) does not affect stroke kinematics at submaximal intensities. Accordingly, the energetic advantage of short course pool should be attributed to turn mechanics rather than to kinematic adjustments during clean swimming.

From an energetic perspective, the contribution of E_lat_ is negligible during predominantly aerobic efforts (Pendergast et al. 2003; Zamparo et al. 2005, 2011, 2020) and our data show that E_aer_ accounts for 99% of E_tot_ in both SC and LC at v_70%mean_, and 97% of E_tot_ at v_86%mean_ in both conditions. Therefore, at intensities below the lactate threshold, performance can be explained almost entirely by aerobic energy pathways (Zamparo et al. 2020). Within this predominantly aerobic domain, our results indicate that, at matched velocities, the overall energy cost of swimming is larger in LC than in SC. This suggests that if swimmers were to maintain in LC the same v_mean_ reached in SC, they would necessarily experience higher physiological and perceptual demands, as sustaining a higher v_clean_ would be required to compensate for the reduced number of turns, which otherwise would reduce v_mean_. From a training methodology point of view, this finding is highly relevant because, to elicit the same physiological responses, swimmers must adopt different v_mean_ depending on pool course with specific temporal differentials. This implies that coaches should be cautious when prescribing similar paces in SC and LC, as they may elicit different physiological responses. Although the only study directly comparing SC and LC did not evaluate energetic parameters at standardized paces between pool courses (Keskinen et al. 2007), it reported SC to be physiologically more advantageous due to the greater number of turns. However, the design of their protocol makes it difficult to isolate the specific effect of turns. In our study, by controlling for both v_clean_ and v_turn_, the differences in performance at a given physiological load were, indeed, explained by the turn segments themselves.

Mean values of C in both SC and LC were consistent with those previously reported in swimmers of comparable level (Capelli et al. 1998; Gonjo et al. 2018; Zamparo et al. 2005). In the literature, C typically increases with velocity according to a quadratic rather than a linear trend. In the present study, however, this relationship appeared linear, likely because C was estimated within a narrow velocity range (≈ 1.1–1.4 m·s⁻¹), where a linear approximation is plausible. When C was extrapolated from the C–v_mean_ relationship at paired velocities, higher values were observed in LC (+ 4.0%). Conversely, when C was assessed at the same relative intensities, v_mean_ was lower in LC (− 5.2%). The consistent differences in C and v_mean_ between SC and LC suggest that the selected intensities were well matched across pool lengths, ensuring that internal load was comparable because it was produced by an equivalent external load. Taken together, these results indicate that, in aerobic swimming performances over the 400 m distance, the eight additional turns in SC compared to LC increase average swimming velocity by approximately 5% at the same overall energy cost, while reducing the overall energy cost by approximately 4% at the same average speed.

It must be pointed out that the 4% difference in energy cost between SC and LC was calculated “neglecting” the scatter of the data (e.g. not considering the inter and intra-individual variability). The large standard deviations in C are essentially attributable to the scatter in the oxygen uptake values, as assessed by means of the back-extrapolation technique. This method has, indeed, a limited precision and, in the literature, it is generally utilized to determine average (group) values rather than individual ones (e.g. Zamparo et al. 2000, 2005). On the other hand, since in this study we wanted to analyze the effect of turns, this prevented us to utilize other methods (such as a continuous monitoring of oxygen uptake), which are more precise but unfeasible in our experimental conditions.

### Limitations

The main limitation of this study concerns the ecological validity of our 400 m trials. Under racing and training conditions, it is unlikely that a swimmer maintains a constant v_mean_ throughout the entire distance. From an energetic point of view, pacing strategies such as the inverted-J or U-shaped profiles were observed during competitions or training sessions (Fang et al. 2024; McGibbon et al. 2018). Accordingly, the imposition of standardized v_turn_ and s_turn_ across laps performed at the same v_mean_ does not accurately replicate the conditions of competitive swimming. This methodological constraint could alter the natural distribution of effort, although evidence indicates that, in predominantly aerobic events, within-race variability of turn is minimal (Polach et al. 2021). Finally, although beyond the scope of this study, most competitive events are performed at higher intensities (Gastin 2001; Seifert and Chollet 2011), highlighting the need for further research to understand the impact of turns at supramaximal intensities.

Additionally, to further enhance the understanding of the impact of turns on C, future studies are warranted to compare trials performed in the pool (with turns) with trials without turns (for example, in a swimming flume or a circular swimming pool).

## Conclusions

The higher velocity consistently observed in short-course compared with long-course swimming races highlights the critical role of turns, which distinguish the two competitive settings. In the present study, the energetic demands attributable to turns during predominantly aerobic performances was quantified for the first time. The addition of turns resulted in a reduction in overall energy cost (− 4%), associated to the higher velocity observed when turns are included (+ 5.2%).

## Data Availability

Raw data are available from the corresponding author on reasonable request.

## Abbreviations

C: Overall energy cost
Ė: Metabolic power
E_aer_: Aerobic energy
E_anL_: Anaerobic lactic energy
E_tot_: Total energy expenditure
LC: Long course pool (50 m)
SC: Short course pool (25 m)
SF: Stroke frequency
s_turn_: Distance of a single turn
t_turn_: Time of a single turn
t_100%turn_: Time of a single turn during v_100mean_
t_XX%turn_: Time of a single turn during XX% of v_100mean_
t_mean_: Time of a single trial
t_100%mean_: Time of a single trial during v_100mean_
t_XX%mean_: Time of a single trial during XX% of v_100mean_
t_clean_: Time of a single lap excluding turn
t_100%clean_: Time of a single lap excluding turn during v_100mean_
t_XX%clean_: Time of a single lap excluding turn during XX% of v_100mean_
v_mean_: Mean swimming velocity
v_100%mean_: Mean swimming velocity at 96% of long-course 400 m best time
v_XX%mean_: XX% of v_100mean_
v_turn_: Mean velocity of the turn
v_100%turn_: Mean velocity of the turn during v_100mean_
v_XX%turn_: XX% of v_100turn_
v_clean_: Mean swimming velocity excluding turns
v_100%clean_: Mean swimming velocity excluding turn during v_100mean_
v_XX%clean_: XX% of v_100clean_
V̇O₂: Oxygen uptake–oxygen uptake at the end of the trial
V̇O₂_net_: Net oxygen uptake at steady state
V̇O₂_rest_: Oxygen Uptake measured at rest breath by breath
